# Pangea breakup and northward drift of the Indian subcontinent reproduced by a numerical model of mantle convection

**DOI:** 10.1038/srep08407

**Published:** 2015-02-12

**Authors:** Masaki Yoshida, Yozo Hamano

**Affiliations:** 1 Department of Deep Earth Structure and Dynamics Research, Japan Agency for Marine–Earth Science and Technology (JAMSTEC), 2-15 Natsushima-cho, Yokosuka, Kanagawa 237-0061, Japan

## Abstract

Since around 200 Ma, the most notable event in the process of the breakup of Pangea has been the high speed (up to 20 cm yr^−1^) of the northward drift of the Indian subcontinent. Our numerical simulations of 3-D spherical mantle convection approximately reproduced the process of continental drift from the breakup of Pangea at 200 Ma to the present-day continental distribution. These simulations revealed that a major factor in the northward drift of the Indian subcontinent was the large-scale cold mantle downwelling that developed spontaneously in the North Tethys Ocean, attributed to the overall shape of Pangea. The strong lateral mantle flow caused by the high-temperature anomaly beneath Pangea, due to the thermal insulation effect, enhanced the acceleration of the Indian subcontinent during the early stage of the Pangea breakup. The large-scale hot upwelling plumes from the lower mantle, initially located under Africa, might have contributed to the formation of the large-scale cold mantle downwelling in the North Tethys Ocean.

Recent plate reconstruction models, based on detailed geological and geomagnetic datasets, have illustrated the specific processes of both the breakup of the supercontinent Pangea and the subsequent continental drift^1^. During the breakup of Pangea, the Indian subcontinent became isolated from the southern part of Pangea, called Gondwanaland, at around 130 Ma, moved northwards, and eventually collided with Eurasia to form the Himalayas at around 40–50 Ma^2^,^3^,^4^,^5^ (Fig. 1). The reason why the Indian subcontinent moved at such a high speed of up to c. 20 cm yr^−1^ 6,7 remains a controversial issue in geodynamics. Numerical simulation of mantle convection under Earth-like conditions provides a unique approach for clarifying this issue by investigating time-dependent mantle convection. Here, numerical simulation of 3-D mantle convection is reported, considering the supercontinent Pangea, to determine the geodynamic mechanisms of the Pangea breakup, the subsequent continental drift, and the high-speed northward drift of the Indian subcontinent.

The mantle was modelled as a Boussinesq fluid with an infinite Prandtl number and realistic Rayleigh number confined in 3-D spherical shell geometry. Conservation equations for mass, momentum, and energy, which govern mantle convection, and the advection equation for those materials compositionally different from the mantle material, were solved using finite-volume-based mantle convection code^8^,^9^ (see Methods for details). Impermeable, shear-stress-free conditions were imposed on both the top and bottom surface boundaries of the spherical shell. The temperature was fixed at the top surface boundary, whereas adiabatic conditions were imposed on the bottom surface boundary.

The simulations in the present study were performed for a period of 200 Myr (i.e., from 200 Ma to the present) to study the behaviour of continental drift and the pattern of mantle convection when the supercontinent Pangea, modelled as a collection of chemically buoyant, highly viscous continental blocks, was imposed *a priori* at the beginning of the simulation.

The lowermost part of Earth's present-day mantle is characterized by the two seismically slow domains beneath Africa and the South Pacific^10^,^11^. While we do not know the thermal heterogeneity in the mantle hundreds of millions of years ago, it is reasonable to suppose that it would have differed from that inferred for the present-day mantle. However, in the present study, based on the assumption that the two large-scale upwelling plumes (i.e., thermo-chemical piles) under Africa and the South Pacific^12^,^13^,^14^,^15^,^16^,^17^, the planetary-scale subduction zone^2^,^3^,^18^ and the resulting degree-two thermal structure of the Earth's mantle interior have persisted throughout the last several hundred million years, the initial state of the lateral temperature anomaly in the mantle at 200 Ma was derived from the S-wave global seismic tomography model, S40RTS^19^.

The seismic velocity anomaly from the S40RTS model was converted to a temperature anomaly using results from mineral physics^20^ (Supplementary Fig. 1). The temperature anomaly from the S40RTS model was imposed within the mantle for depths greater than 660 km, and the temperature anomaly in the upper mantle was uniformly set at zero (Supplementary Fig. 2b–f). This is because the thermal heterogeneity in the upper mantle derived from the original seismic tomography model represents the present-day tectonic characteristics, i.e., the configuration of subducting plates as high velocity anomalies, the distribution of plate divergence as low velocity anomalies, and the distribution of neutrally buoyant continental roots (i.e., tectosphere) as high velocity anomalies above c. 300 km^21^.

Following the previous work^9^, the geometry of the modelled Pangea was taken from the digital data of the coastline of Pangea at 200 Ma^1^. Pangea was initially broken up into seven major continental blocks (i.e., Eurasia, Africa, North America, South America, Antarctica, Australia, and the Indian subcontinent; Supplementary Fig. 2a), and the continental boundaries were treated as a narrow oceanic mantle. The thickness of Pangea was initially set to 202 km. The density difference between the chemically buoyant Pangea and the ambient mantle was taken to be −100 kg m^−3^^22^,^23^. The viscosity ratio between the highly viscous Pangea and the ambient mantle (*Δη_C_*) was taken as a free parameter, and it was varied from 10^2^ to 10^4^ in the present study. Considering the thermal insulation effect of the supercontinent, as suggested by several numerical models^24^,^25^,^26^,^27^,^28^,^29^,^30^,^31^, the temperature of the subcontinental mantle beneath Pangea, from depths of 202–660 km, was assumed to be *ΔT_e_* higher than that of the mantle beneath oceanic regions at the same depths (see the Discussion section for further details on the thermal insulation effect). In the present study, *ΔT_e_* was taken to be +200 K (or lower for some models).

To study the mantle dynamics associated with the breakup of Pangea, previous work has imposed rigid continental blocks in 3-D spherical models of mantle convection and has solved continental drift using a kinematic approach^32^. In contrast, our numerical model allows the highly viscous continental material to deform and drift independently under the convective force of the ambient mantle^8^,^9^.

## Results

The models performed in the present study are listed in Table 1. The main free parameter was the viscosity ratio between the highly viscous lithosphere of Pangea and the ambient mantle, *Δη_C_*; other free parameters were the viscosity contrast between the top surface and the underlying mantle due to the temperature-dependent viscosity, *Δη_T_* (see Methods), and the excess temperature anomaly of the subcontinental mantle beneath Pangea, *ΔT_e_*.

**Figure 2:**
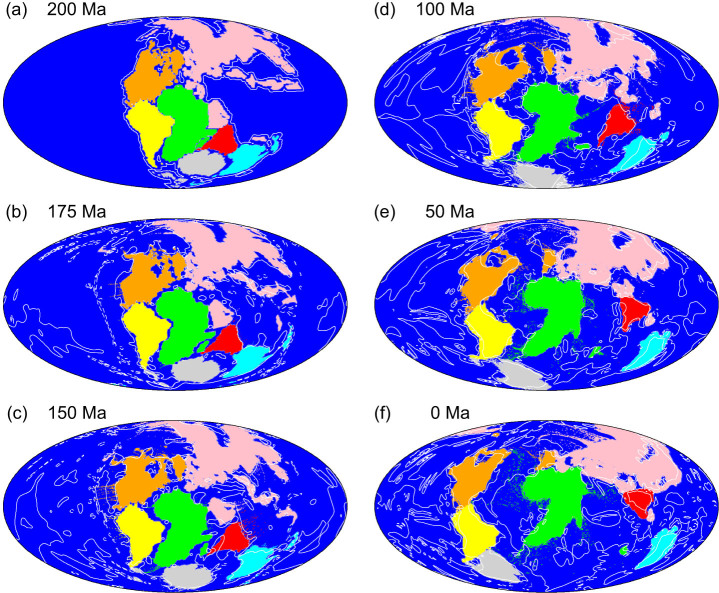
Time sequence of the positions of drifting continents for Model C30 with a viscosity ratio between Pangea and the ambient mantle (*Δη_C_*) of 10^3^. The Indian subcontinent is indicated in red. White contour lines show the temperature anomaly (i.e., the deviation from the horizontally averaged temperature) of the upper mantle at a depth of 358 km. Contour intervals are 250 K. Solid and dashed lines represent positive and negative temperature anomalies, respectively. This map was produced using the Generic Mapping Tools^56^.

**Figure 3:**
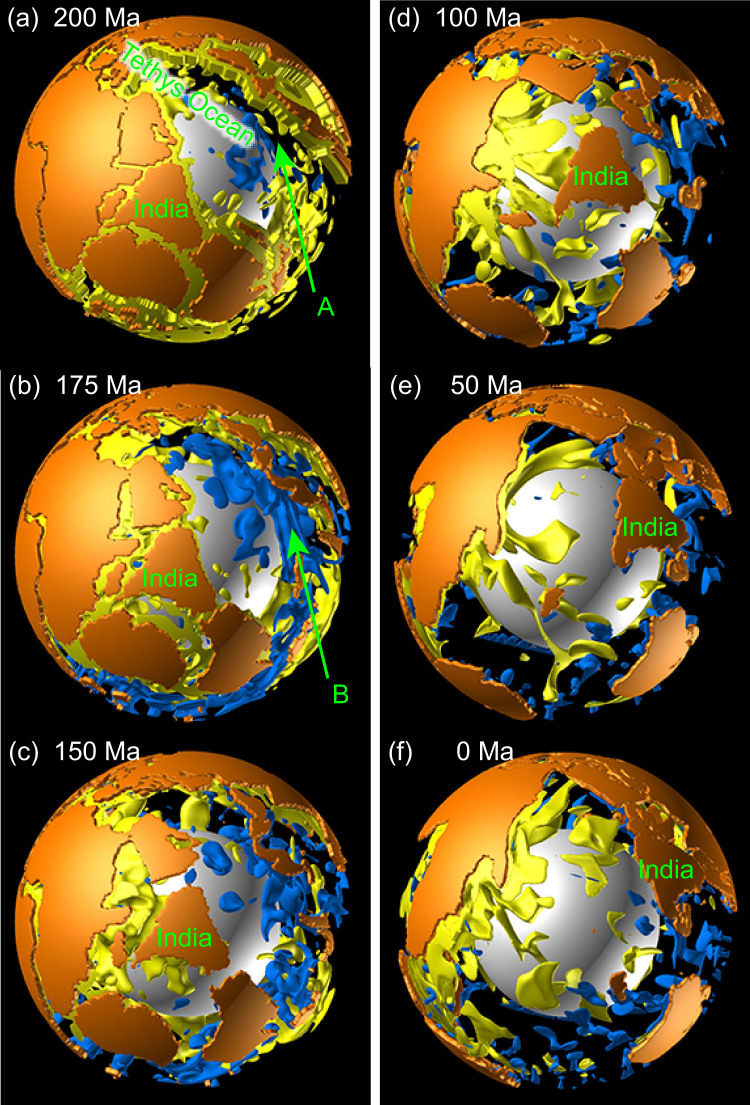
Time sequence of 3-D views of mantle convection and drifting continents for Model C30 with *Δη_C_* = 10^3^. The cyan and yellow isosurfaces of the temperature anomaly (i.e., the deviation from the laterally averaged temperature at each depth) indicate −250 K and +100 K, respectively. The isosurfaces of the temperature anomaly at depths of less than 202 km are omitted for clarity. Positions of the continents are represented by orange regions. White sphere indicates the bottom of the mantle. This figure was produced using the commercial visualization software AVS/Express.

Figure 2 shows the time sequence of the position of drifting continents for Model C30 with *Δη_C_* = 10^3^, which ensures dynamic stability of the drifting continents^33^,^34^,^35^,^36^. Figure 3 shows the time sequence for 3-D views of mantle convection and drifting continents for Model C30. The modelled Pangea broke up and each continental block moved with time following the initiation of the simulation. The “present-day” continental distribution at 0 Ma obtained in this simulation roughly resembles the present-day continental distribution of the real Earth, although certain blocks (i.e., Antarctica) are not located in their true present-day locations (Fig. 2f).

**Figure 4:**
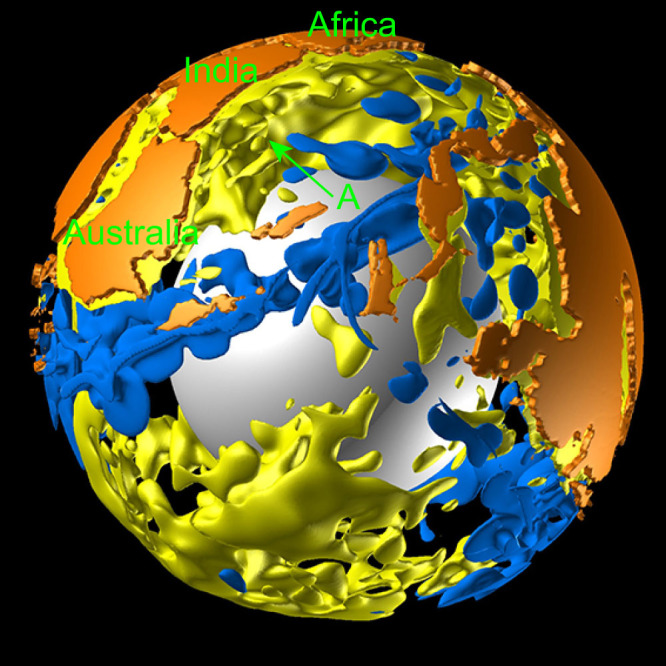
This figure shows Figure 3b from a different viewpoint. This figure was produced using the commercial visualization software AVS/Express.

The most notable feature of this result is that the Indian subcontinent moved towards the north at high speed following its separation from Gondwanaland, before it eventually collided with Eurasia (Fig. 2f). Our numerical simulation revealed that a major factor in the high-speed northward drift of the Indian subcontinent was the large-scale cold mantle downwelling that developed spontaneously in the North Tethys Ocean (B in Fig. 3b), attributed to the overall shape of the supercontinent Pangea. This mantle downwelling flow was minor in the initial state of the temperature anomaly in the mantle (A in Fig. 3a). These cold downwellings originated from the subduction of surface oceanic mantles, which eventually joined with the cold suboceanic mantle initially present in this region (Supplementary Fig. 2c–f). This numerically-obtained large-scale downwelling in the North Tethys Ocean region is consistent with the subduction history of the Indian region compiled by both seismic tomography and plate reconstruction^37^. In addition, a global plate reconstruction model demonstrated that the age of oceanic lithosphere in the North Tethys Ocean was c. 180 Ma, the oldest ocean floor in the world of the day^1^, which means that mantle downwellings developed preferentially from this region. The large-scale hot upwelling plumes from the lower mantle, initially located under Africa (A in Fig. 4), might have contributed to the formation of the large-scale cold mantle downwelling in the North Tethys Ocean.

The northward drift of the Indian subcontinent observed in Fig. 2 is a robust feature observed in all of the models of the present study. When *Δη_C_* = 10^2^ (i.e., one order of magnitude smaller than for the model shown in Fig. 2) and 10^2.5^ (Models C20 and C25, respectively), the Indian subcontinent still moved northwards and collided with Eurasia, although all the continental blocks were considerably deformed over time because of their dynamical weakness (Supplementary Figs. 3 and 4). On the other hand, when *Δη_C_* = 10^4^ (i.e., one order of magnitude larger than for the model shown in Fig. 2) and 10^3.5^ (Models C40 and C35, respectively), continental blocks were less deformed by mantle convection because the advection of the continental material was decoupled from the ambient mantle convection. Therefore, the time required to move the Indian subcontinent and to reproduce the present-day continental distribution was much longer. Thus, continental drift is not realized within the time scale of the real Earth when *Δη_C_* is larger than 10^3^ (Supplementary Figs. 5 and 6).

**Figure 5:**
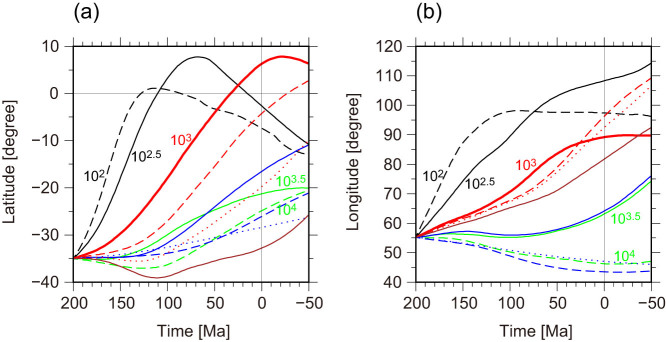
Time tracking of the “centre” of the Indian subcontinent for 250 Myr from 200 Ma along (a) latitudinal and (b) longitudinal directions for the no-temperature-dependent-viscosity models with *Δη_C_* = 10^2^ (black dashed lines; Model C20), 10^2.5^ (black solid lines; Model C25), 10^3^ (red solid lines; Model C30), 10^3.5^ (green solid lines; Model C35), and 10^4^ (green dashed lines; Model C40); for the temperature-dependent models with *Δη_C_* = 10^3^ and *Δη_T_* = 10^1^ (blue solid lines Model C30T10), *Δη_C_* = 10^3^ and *Δη_T_* = 10^2^ (blue dashed lines; Model C30T20), and *Δη_C_* = 10^3^ and *Δη_T_* = 10^3^ (blue dotted lines; Model C30T30); and for the models with *Δη_C_* = 10^3^ and an excess temperature anomaly beneath Pangea of *ΔT_e_* = +100 K (red dashed lines; Model C30H100), +50 K (red dotted lines; Model C30H050), and 0 K (i.e., no excess temperature anomaly; brown solid lines; Model C30H000).The “centre” is defined at the location of −35°N and 50°E at 200 Ma.

Figure 5 shows the time tracking of the “centre” of the Indian subcontinent for 250 Myr from 200 Ma along latitudinal and longitudinal directions for the models examined in the present study. This result quantitatively revealed that the trace of the Indian subcontinent was sensitive to the choice of *Δη_C_*, and, when *Δη_C_* = 10^3^, the Indian subcontinent gradually accelerated before it collided with Eurasia, and then slowed down after the collision event.

Here, we demonstrate the results of additional simulations using a numerical model of mantle convection with temperature-dependent viscosity to show the dynamic effects of a laterally viscosity heterogeneity on the Pangea breakup and subsequent continental drift. The activation parameter, *E* ≡ ln(*Δη_T_*), that controls the viscosity contrast between the top surface and the underlying mantle (see Eq. (7) in Methods), was set to 2.30 (i.e., *Δη_T_* = 10^1^; Model C30T10), 4.61 (*Δη_T_* = 10^2^; Model C30T20), and 6.91 (*Δη_T_* = 10^3^; Model C30T30) in the present study.

Figure 5 shows that the Indian subcontinent slowed down with increasing *Δη_T_* (see the three blue lines in Fig. 5) because the high viscosity of the top surface thermal boundary layer blocked the acceleration of the continental drift on the modelled Earth. This result implies that a yielding rheology (i.e., pseudo visco-plastic rheology) was required to reproduce plate-like behaviour in the highly viscous thermal boundary layer^38^,^39^,^40^, which reproduces the variety of “plate boundary forces” acting on the continental margins^41^.

**Figure 6:**
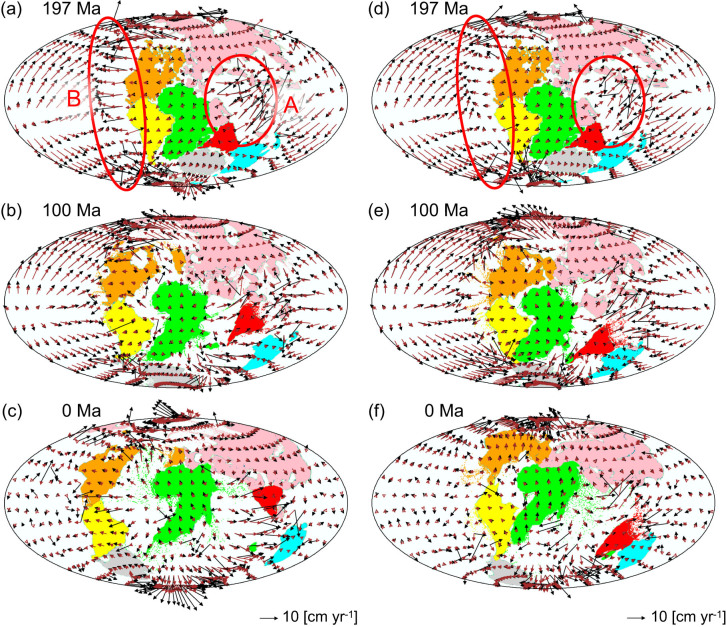
Time sequence of the positions of drifting continents and the velocity fields at the top surface (black arrows) and at a depth of 302 km (brown arrows) for the model with *Δη_C_* = 10^3^, and with (a to c; Model C30) and without (d to f; Model C30H000) the high temperature anomaly beneath Pangea. This map was produced using the Generic Mapping Tools^56^.

Figure 6 illustrates the effects of the high-temperature anomaly (HTA) region due to the thermal insulation effect beneath Pangea, by showing the time sequence of the drifting continents and velocity fields at the top surface boundary and at a depth of 302 km for the model with *Δη_C_* = 10^3^, both with (Fig. 6a–c; Model C30) and without (Fig. 6d–f; Model C30H000) the HTA region. When the HTA region beneath Pangea is considered, the speed of the lateral flow of mantle around Pangea is fast during the early stage of the Pangea breakup (i.e., 197 Ma), compared with the model that does not include the HTA region (cf. red circles in Fig. 6a and d). In the absence of the HTA region, the Indian subcontinent eventually moved towards the north, but it did not reach Eurasia in the Northern Hemisphere (Fig. 6e and f). As a result, the “present-day” continental distribution at 0 Ma exhibits less similarity to that of the present-day continental distribution of the real Earth, compared with the model that included the HTA region (Fig. 6f, see also Supplementary Fig. 7). This result demonstrates that the strong lateral mantle flow caused by the HTA region beneath Pangea (A in Fig. 6a) enhanced the acceleration of the Indian subcontinent during the early stage of the Pangea breakup. In addition, the strong lateral mantle flow caused by the HTA region (B in Fig. 6a) served as the driving force behind the westward drift of North and South America, which led to the formation of the Atlantic Ocean, elongated from north to south.

When the temperature anomaly of the subcontinental mantle beneath Pangea, *ΔT_e_*, decreased below +200 K, the time required to move the Indian subcontinent and to reproduce the present-day continental distribution increased significantly (Model C30H100 with *ΔT_e_* = +100 K, Model C30H050 with *ΔT_e_* = +50 K, and Model C30H000 with *ΔT_e_* = 0 K, shown as dashed and dotted red lines and solid brown lines in Fig. 5, respectively). On the other hand, when *ΔT_e_* was +200 K and the lateral temperature anomaly in the lower mantle, which was derived from the S40RTS tomography model, was not considered (i.e., *λ* = 0 in Eq. (11), see Methods), the Pangea breakup and subsequent continental drift appears to be realized within the time scale of the real Earth. However, the Indian subcontinent and Africa stuck together over time and the “present-day” continental distribution at 0 Ma obtained in this simulation did not resemble the actual present-day continental distribution (Supplementary Fig. 8). These results suggest that the existence of both the HTA region in the upper mantle and the lateral temperature anomaly in the lower mantle are required for the formation of the present-day continental distribution.

## Discussion

One of the major findings of the present study is that the degree of deformation of continents depends largely on the value of *Δη_C_* and that the stable continental drift observed in the real Earth is realized under severely limited *Δη_C_*. This implies that the drag force due to the non-uniform lateral mantle flow acting on the base of the continents increases the local deformation of the continental material, which makes it difficult to move the continents rigidly. In fact, when *Δη_C_* is small (e.g., *Δη_C_* ≤ 10^2.5^), the greater the continent's size, the larger the degree of continental deformation is (Supplementary Figs. 3 and 4). When the value of *Δη_C_* is a moderate value for realizing realistic continental drift with minimal deformation (*Δη_C_* = 10^3^ in the present model; Fig. 2), the speed of flow in the subcontinental mantle is close to the speed of continental drift (Fig. 6a–c). In this case, when the size of the continent is small, the drag force acting on the base of the continent is relatively uniform such that the continental block moves with only small deformation. This is an important reason why the small Indian subcontinent moved at high speed without considerable deformation.

A previous numerical study of mantle convection models with temperature-, phase-, and composition-dependent viscosity and yield stress (i.e., pseudo visco-plastic rheology) revealed that the behaviour of continental drift following the breakup of Pangea is very sensitive to the magnitude of yield stress, and suggested that the actual continental drift of the Earth is accomplished by mantle convection within limited geophysical conditions^9^. However, for the simulation approach used in this study, the subsequent pattern of drifting continents depends on the initial mantle convection pattern. The dynamic role of lateral viscosity heterogeneity in the Pangea breakup and the subsequent continental drift remains to be explained in future studies. In particular, an understanding of the effects of plume heat flux on the acceleration and deceleration of the Indian plate before its collision with Eurasia is required, using future 3-D numerical models with temperature-dependent rheology of mantle materials, as suggested by a previous numerical model^5^.

Our systematic case study suggested that the existence of the widespread HTA region beneath Pangea, due to the thermal insulation effect, enhanced the acceleration of the Indian subcontinent during the early stage of the Pangea breakup. Indeed, whether such a HTA region was widespread beneath Pangea is a controversial issue in geoscience^27^ ever since the hypothesis by Don L. Anderson who proposed that the breakup of Pangea would be caused by a temperature increase beneath Pangea due to the thermal insulation effect^29^. This was supported by several numerical studies of mantle convection^24^,^25^,^26^,^27^,^28^,^29^,^30^,^31^, whereas other numerical studies insisted that there was no significant temperature increase caused by the continental assembly^42^,^43^. This latter conflicting result was due to the relative dominance of cold downwelling in mantle convection, which causes a shorter convection wavelength and a lower mantle temperature associated with a decrease in heat transport efficiency^44^. Our model simply assumed that the widespread temperature increase beneath Pangea existed which in our modelling (Supplementary Fig. 2b) caused the continental breakup and may underlie intermittent eruption of continental flood basalts^4^,^27^,^45^. However, there is a possibility that the temperature anomaly beneath Pangea may be spatially non-uniform due to the active subduction of oceanic plates along the margins of Pangea. The effect of the spatially non-uniform HTA region on the breakup of Pangea and the subsequent continental drift should be investigated carefully in future.

To summarize the results of the present study, it was determined that the high-speed northward drift of the Indian subcontinent was likely caused by large-scale mantle downwelling that developed spontaneously in the North Tethys Ocean, attributed to the overall shape of the supercontinent Pangea. This result implies that the high-speed northward drift of the Indian subcontinent and the resulting Indo-Asian convergence at around 40–50 Ma was destined when the supercontinent Pangea was formed at around 300 Ma.

It is about one hundred years since the publication of the first edition of “*Die Entstehung der Kontinente and Ozeane* (*The Origin of Continents and Oceans*)”^46^ by Alfred Wegener, who first proposed the theory of continental drift in 1912. Now, we can approximately reproduce the process of continental drift, from the breakup of Pangea at about 200 Ma to the present-day continental distribution, using a numerical simulation model of 3-D spherical mantle convection with an infinite Prandtl number and an Earth-like Rayleigh number. Arthur Holmes, a British geologist, first proposed that the cause of continental drift was subcrustal convection currents within the Earth^47^. Our numerical simulations demonstrated that toroidal flow of the surface boundary layer, generated by the lateral viscosity heterogeneity between the highly viscous continents and the oceanic mantle, played a significant role in the modelled Pangea breakup and implied that the primary driving force of continental dispersal was both mantle convection (i.e., large-scale mantle upwelling/downwelling flows) and plate tectonics (i.e., plate boundary forces).

## Methods

The mantle was modelled as a Boussinesq fluid with an infinite Prandtl number and realistic Rayleigh number confined in 3-D spherical shell geometry (*r*, *θ*, *ϕ*) with a thickness of 2867 km. Mantle convection was numerically solved using the finite-volume-based mantle convection code, ConvGS^8^,^9^. The number of computational grids was taken to be 128 (*r*) × 128 (*θ*) × 384 (*ϕ*) × 2 (for Yin and Yang grids^48^). Impermeable, shear-stress-free conditions were imposed on both the top and bottom surface boundaries of the spherical shell. The temperature was fixed at the top surface boundary, whereas adiabatic conditions were imposed on the bottom surface boundary.

The dimensionless conservation equations for mass, momentum, and energy, which govern mantle convection under the Boussinesq approximation, and the advection equation for those materials compositionally different from mantle material, were expressed, respectively, as:


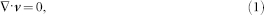

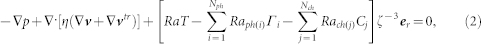

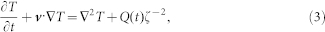

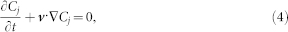
where ***v*** represents the velocity, *p* the dynamic pressure, *η* the viscosity, *t* the time, *T* the temperature, *Q* the time-dependent radioactive heat production rate per unit mass, *Γ_i_* the phase function (0 ≤ *Γ_i_* ≤ 1)^49^, *C_j_* the composition (0 ≤ *C_j_* ≤ 1), *i* the index of each phase in the mantle, *j* the index of each material compositionally different from mantle material, and ***e****_r_* the unit vector in the upward radial direction.

The dimensionless parameters were the thermal Rayleigh number *Ra*, the phase Rayleigh number *Ra_ph_*_(*i*)_, the compositional Rayleigh number *Ra_ch_*_(*j*)_, the internal heating number *Q*, and the mantle/shell radius ratio *ζ*: 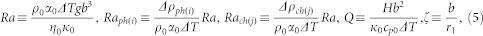
where the definitions and values of the symbols are as listed in Supplementary Table 1.

The thermal Rayleigh number *Ra* was fixed at 5.72 × 10^7^ (corresponding to a reference viscosity of 10^21^ Pa s and a temperature difference across the mantle of 2500 K). The internal heat production depends on time, considering the decay of radioactive elements in the chondritic mantle^9^,^50^. The olivine to wadsleyite phase transition at a reference depth of 410 km, the wadsleyite to ringwoodite phase transition at a depth of 520 km, and the ringwoodite to perovskite + magnesiowüstite phase decomposition at a depth of 660 km were imposed in the model (see Supplementary Table 1).

The viscosity of the mantle in the present model depends on the phase and composition according to a dimensionless formulation:
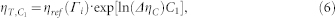
where *C*_1_ is the composition that represents continental (*C*_1_ = 1) and mantle (*C*_1_ = 0) materials, and *Δη_C_* is the viscosity ratio between the continental and mantle materials, which is taken as a free parameter. The reference viscosity at each phase *η_ref_*(*Γ_i_*_ = 1,2_) is set to 1 in the olivine, wadsleyite, and ringwoodite phases, and *η_ref_*(*Γ_i_*_ = 3_) is set to 30 in the perovskite + magnesiowüstite phase^51^,^52^,^53^.

Note that the temperature-dependence of viscosity was not considered in the present model because activation parameters of rocks in the deep mantle have not yet been determined, even in recent mineral physics experiments^20^. Notwithstanding, in the present study, we also obtained results from additional simulations with a numerical model of mantle convection using a temperature-dependent viscosity, to show the dynamic effect of laterally variable rheology on the Pangea breakup and the subsequent continental drift.

For the temperature-dependent viscosity model, Eq. (6) was replaced by
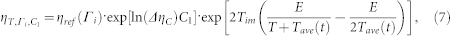
where *T_ave_*(*t*) is the dimensionless average temperature of the entire mantle at each time step, and *E* ≡ ln(*Δη_T_*) is the activation parameter that controls the viscosity contrast between the top surface and the underlying mantle.

Based on the half-space cooling model^54^, the initial condition for the depth profile of dimensionless temperature is given by 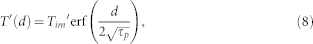
where the prime indicates a dimensionless value, *T_im_*' is the dimensionless initial mantle temperature of 0.52 (corresponding to 1573 K), *d* is the depth (i.e., *d* = 0 and 1 at the top and bottom boundaries, respectively), 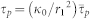, and 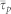 is set at the typical age of the lithosphere (100 Myr).

In the present model, we assumed that the seismic velocity perturbation depends on the temperature perturbation alone. The initial state of the lateral temperature anomaly in the mantle at 200 Ma was derived from the S-wave global seismic tomography model, S40RTS^19^. The seismic velocity anomaly *δV_s_* from the S40RTS model was converted to the temperature anomaly *δT*: 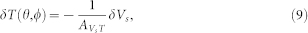
where *δV_s_* is the deviation from the PREM^55^, and the temperature derivative of the seismic velocity anomaly as a function of depth obtained by mineral physics experiment^20^ (Supplementary Fig. 1): 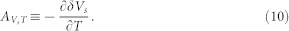
In addition, considering the thermal insulation effect of the supercontinent^24^,^25^,^26^,^27^,^28^,^29^,^30^, the temperature of the subcontinental mantle beneath Pangea from depths of 202 to 660 km is assumed to be *ΔT_e_* higher than that of the mantle beneath the oceanic regions at the same depths.

Finally, the dimensionless initial temperature field in the mantle at the beginning of the simulation was 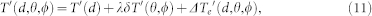
where *λ* is 0 or 1, and *ΔT_e_*' is the dimensionless excess temperature anomaly of the subcontinental mantle beneath Pangea. When *λ* is 0, there is no lateral temperature anomaly for the initial temperature field.

The obtained temperature anomaly in the mantle is shown in Supplementary Fig. 2b–f.

## Author Contributions

M.Y. designed the project and carried out the numerical simulations and data analyses. M.Y. and Y.H. discussed the results and prepared the manuscript.

## Supplementary Material

Supplementary Information Supplementary Information

## Figures and Tables

**Figure 1:**
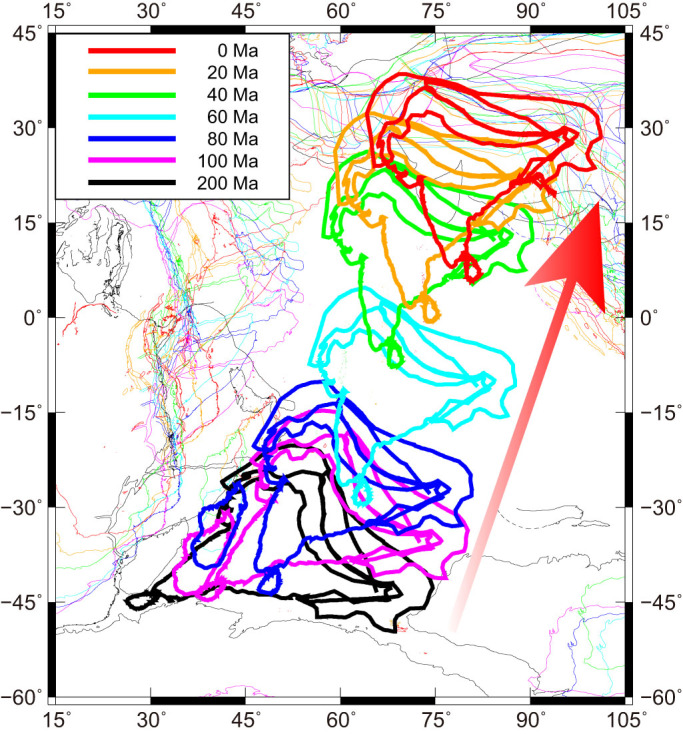
Time sequence of drifting continental blocks at each age from 200 Ma to the present^1^. The Indian subcontinent that moved towards the north and collided with Eurasia is highlighted by thick contour lines. This map was produced using the Generic Mapping Tools^56^.

**Table 1:** Models examined in the present study

Model name	*Δη_C_*	*Δη_T_*	*ΔT_e_* [K]	*λ*^a^	Figure(s)
C30	10^3^	0	+200	1	2, 3, 4, 5, 6
C20	10^2^	0	+200	1	5, S3
C25	10^2.5^	0	+200	1	5, S4
C35	10^3.5^	0	+200	1	5, S5
C40	10^4^	0	+200	1	5, S6
C30NL	10^3^	0	+200	0	S8
C20NL	10^2^	0	+200	0	
C30T10	10^3^	10^1^	+200	1	5
C30T20	10^3^	10^2^	+200	1	5
C30T30	10^3^	10^3^	+200	1	5
C30H000	10^3^	0	0	1	5, 6, S7
C30H050	10^3^	0	+50	1	5
C30H100	10^3^	0	+100	1	5

^a^See Eq. (11); when λ = 0, there is no lateral temperature anomaly in the lower mantle for the initial field.
