# Supplementary material for: Pangea breakup and northward drift of the Indian subcontinent reproduced by a numerical model of mantle convection
Source: Sci Rep. 2015 Feb 12;5:8407. doi: 10.1038/srep08407 (PMC4325333; doi:10.1038/srep08407)
Supplement: Supplementary Information [file srep08407-s1.pdf]

Supplementary Information for

Pangea breakup and northward drift of the Indian subcontinent reproduced by a numerical model of mantle convection

Masaki Yoshida[1] & Yozo Hamano[1]

[1]Department of Deep Earth Structure and Dynamics Research, Japan Agency for Marine–Earth Science and Technology (JAMSTEC), 2-15 Natsushima-cho, Yokosuka, Kanagawa 237-0061, Japan.

Correspondence and requests for materials should be addressed to M.Y. (myoshida@jamstec.go.jp)

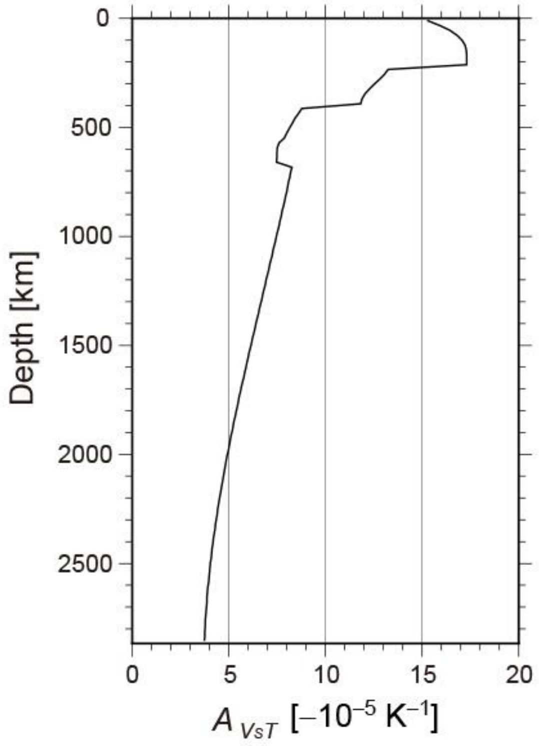

Supplementary Figure 1. Depth profile of the temperature derivatives of the S-wave seismic velocity anomalies, A_{V_sT} (see Eq. (10) in the text).

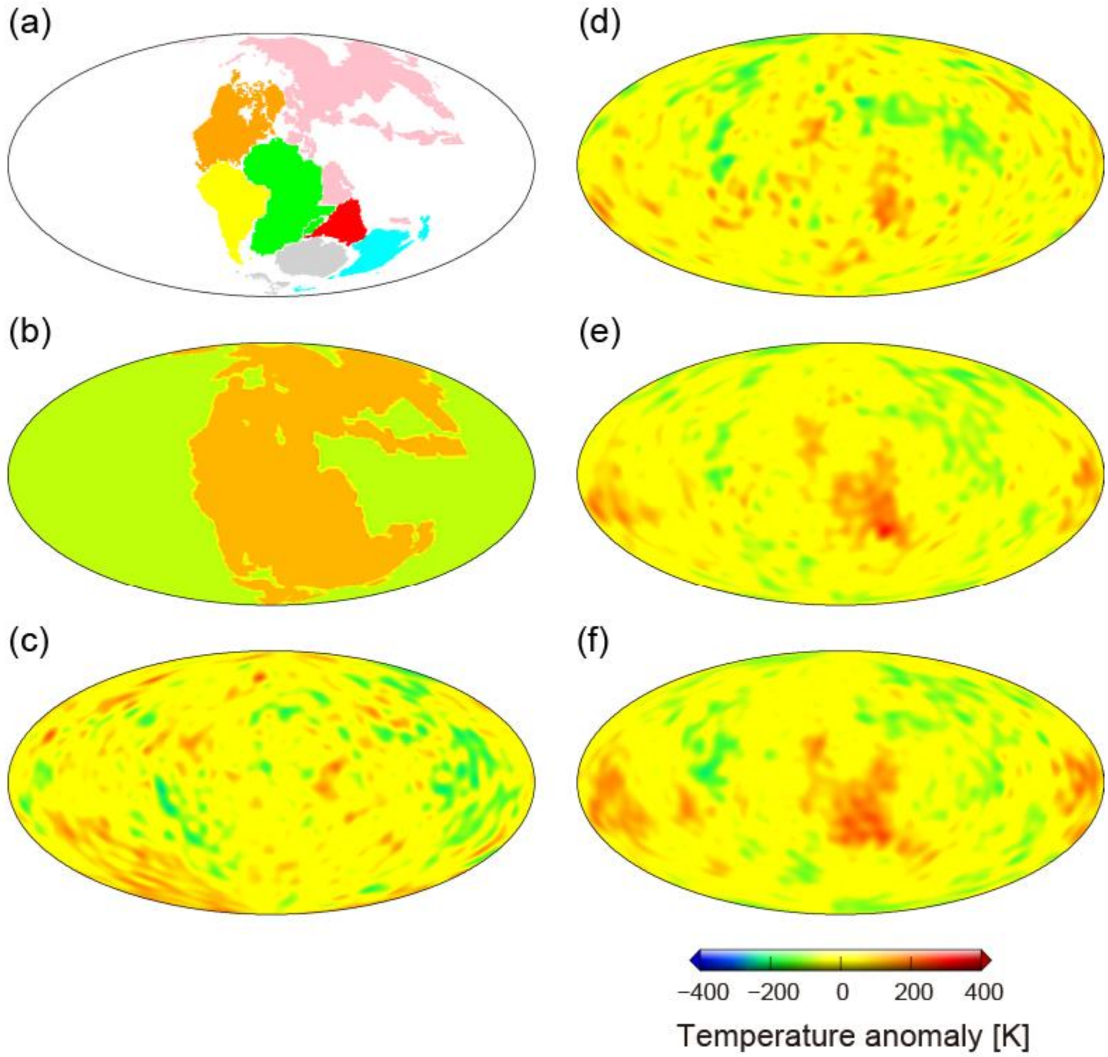

Supplementary Figure 2. (**a**) Distribution of continents at 200 Ma. (**b** to **f**) Initial state of the temperature anomaly at depths of (**b**) 358, (**c**) 717, (**d**) 1433, (**e**) 2150, and (**f**) 2509 km. This map was produced using the Generic Mapping Tools.

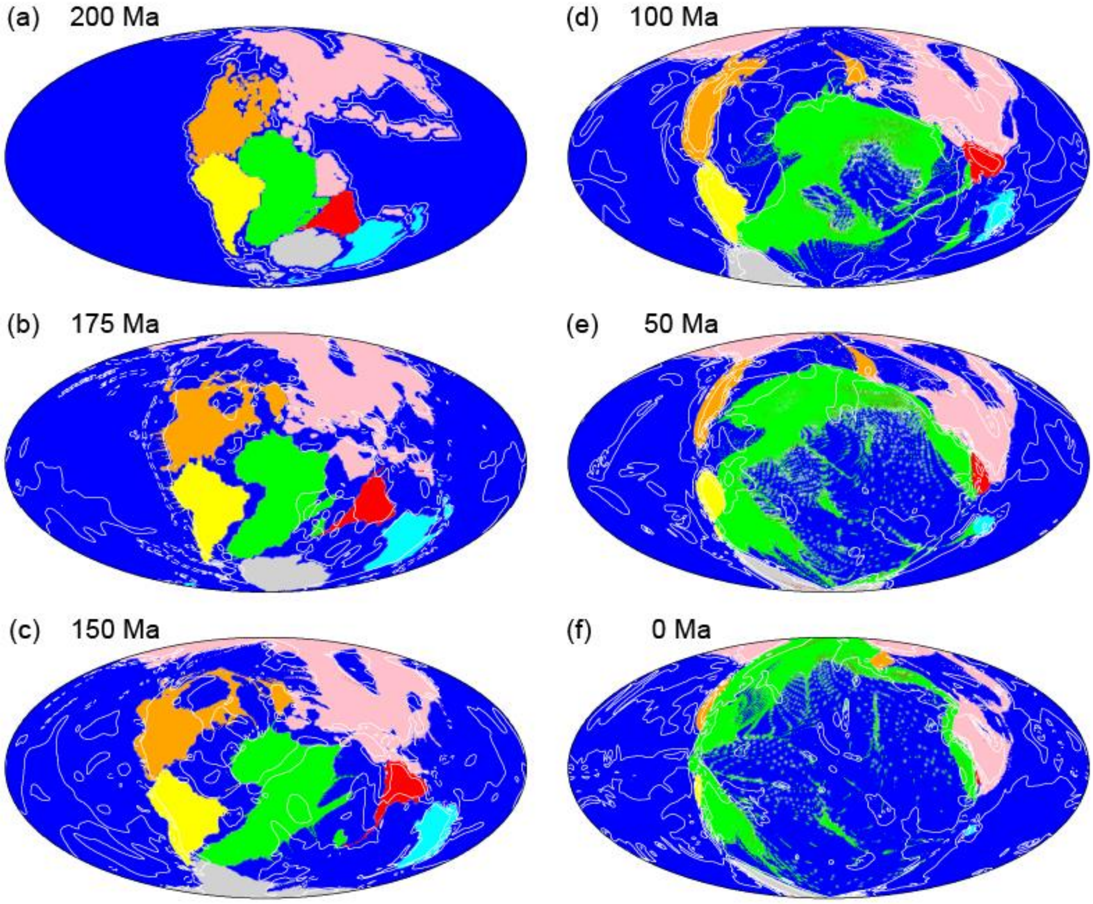

Supplementary Figure 3. Time sequence of the positions of drifting continents for Model C20 with the viscosity ratio of Pangea and the surrounding mantle ($\Delta\eta_C$) of 10^2 and with the high temperature anomaly beneath Pangea. The Indian subcontinent is indicated in red. White contour lines show the temperature anomaly (i.e., the deviation from the horizontally averaged temperature) of the upper mantle at a depth of 358 km. Contour intervals are 250 K. Solid and dashed lines represent positive and negative temperature anomalies, respectively. This map was produced using the Generic Mapping Tools.

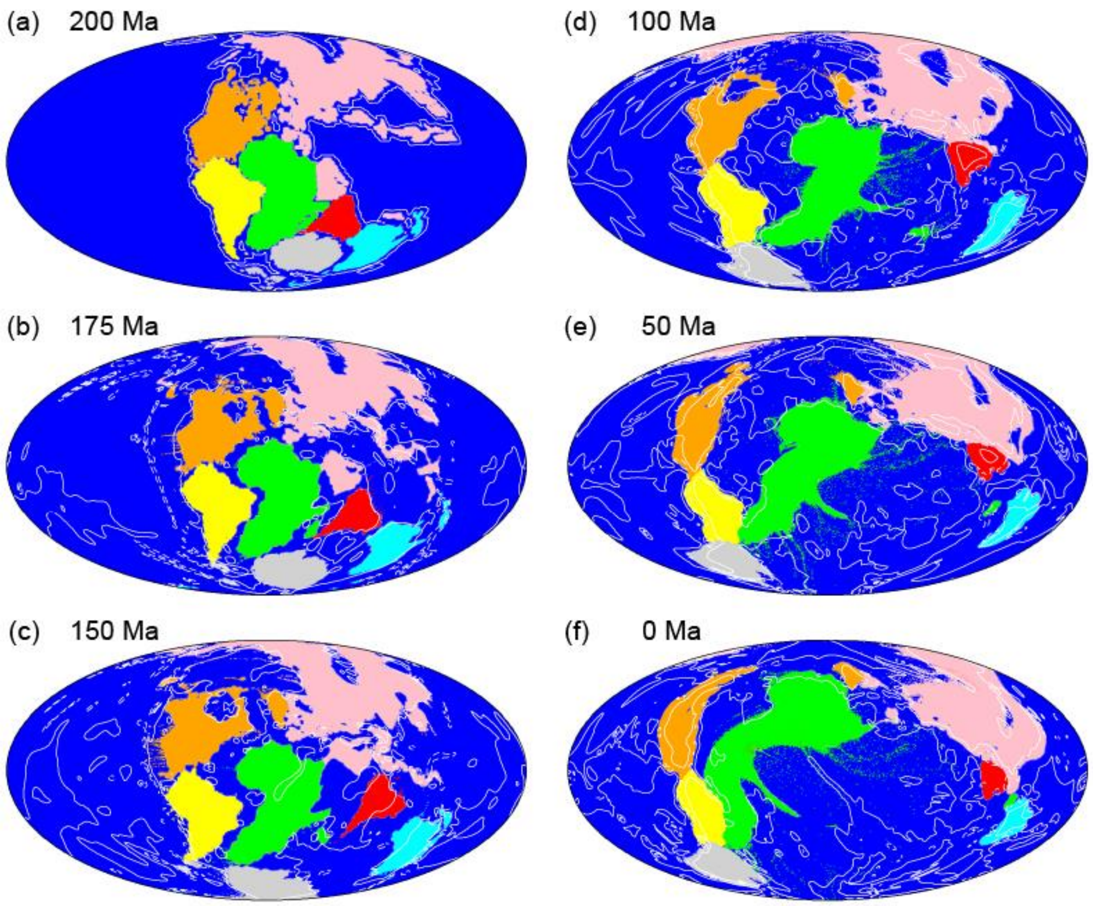

Supplementary Figure 4. Time sequence for Model C25 with $\Delta\eta_C = 10^{2.5}$ (other details are as for Supplementary Fig. 3). This map was produced using the Generic Mapping Tools.

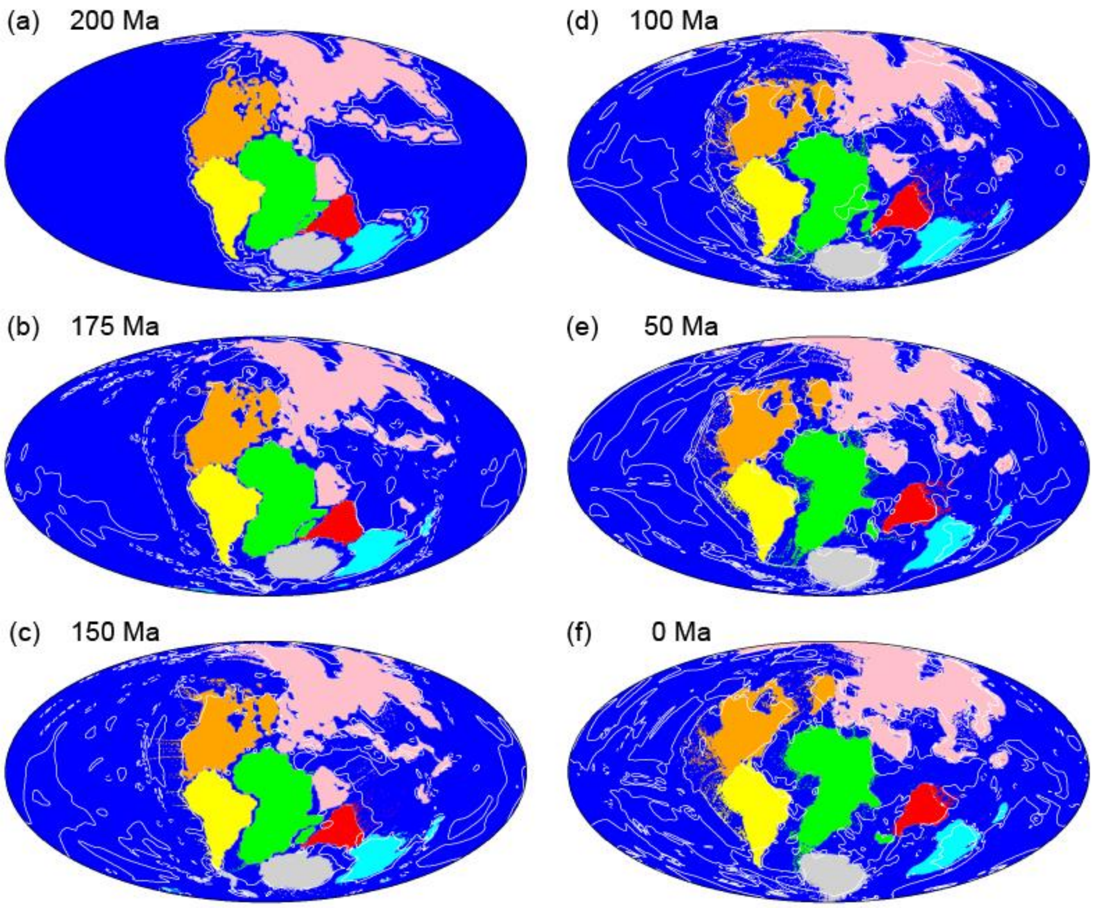

Supplementary Figure 5. Time sequence for Model C35 with $\Delta\eta_C = 10^{3.5}$ (other details are as for Supplementary Fig. 3). This map was produced using the Generic Mapping Tools.

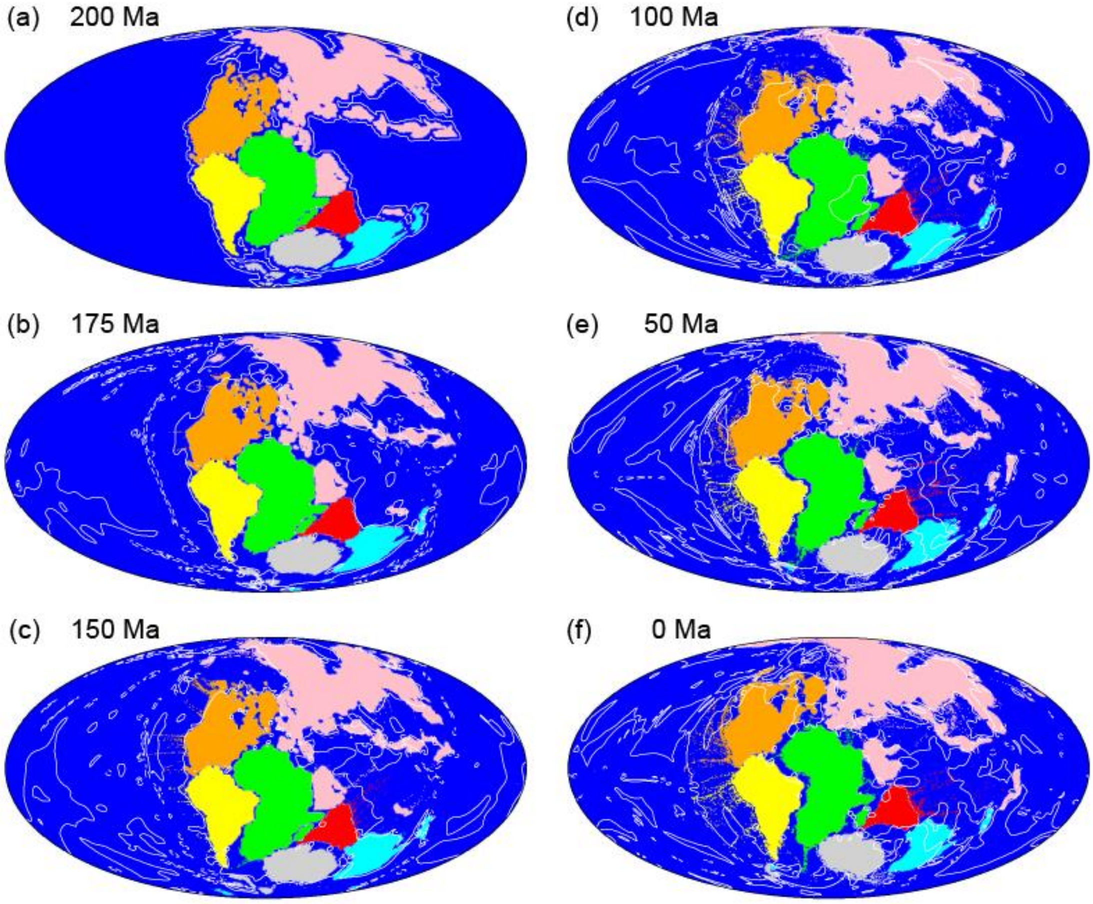

Supplementary Figure 6. Time sequence for Model C40 with $\Delta\eta_C$ = of 10^4 (other details are as for Supplementary Fig. 3). This map was produced using the Generic Mapping Tools.

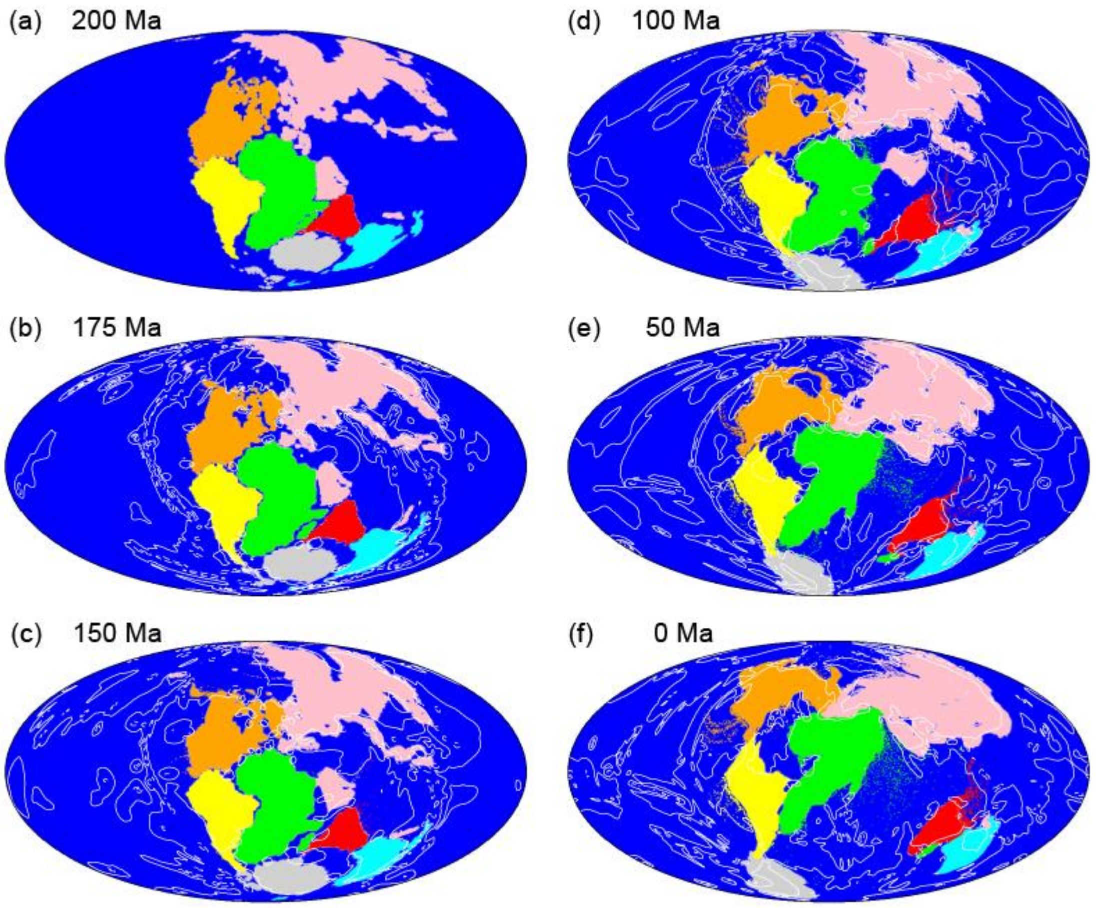

Supplementary Figure 7. Time sequence for Model C30H000 with $\Delta\eta_C$ = of 10^3 and without the high temperature anomaly beneath Pangea (other details are as for Supplementary Fig. 3). This map was produced using the Generic Mapping Tools.

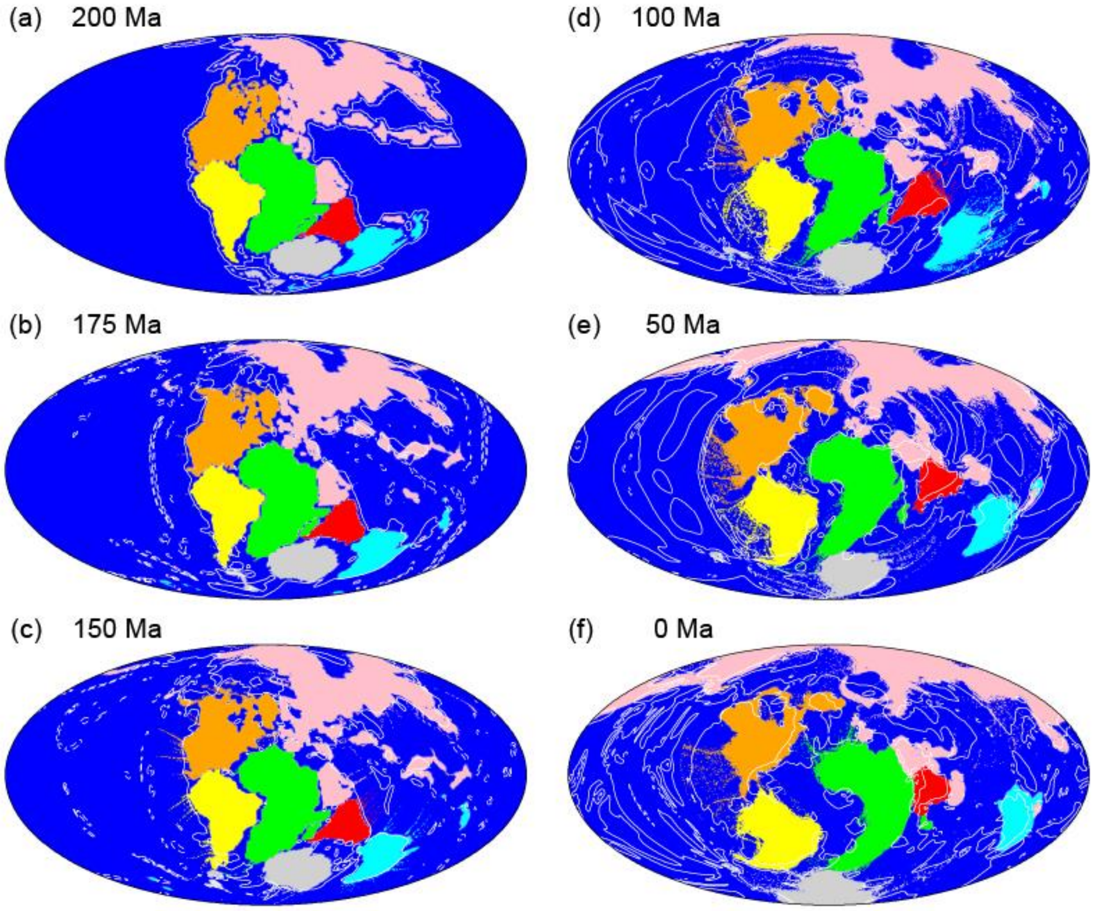

Supplementary Figure 8. Time sequence for Model C30NL with $\Delta\eta_C$ = of 10^3 and without a lateral temperature anomaly in the lower mantle (other details are as for Supplementary Fig. 3). This map was produced using the Generic Mapping Tools.

Supplementary Table 1. Model parameters used in the present study.

Symbol	Definition	Value	Unit
g	Gravitational acceleration	9.81	m s^{-2}
ρ_0	Reference density of mantle	3300	kg m^{-3}
α_0	Reference thermal expansivity of mantle	3 × 10^{-5}	K^{-1}
T_{top}, T_{bot}	Temperatures at the top and bottom	273, 2773	K
ΔT	Temperature difference across the mantle	2500	K
r_1, r_0	Radii of Earth and its core	6371, 3504	km
b	Thickness of mantle	2867	km
κ_0	Reference thermal diffusivity of mantle	10^{-6}	m^2 s^{-1}
η_0	Reference viscosity of upper mantle	10^{21}	Pa s
c_{p0}	Reference specific heat at constant pressure of mantle	1250	J kg^{-1} K^{-1}
H	Radioactive heat production rate per unit mass	Time-dependent	W kg^{-1}
-	Clapeyron slope at 410-, 520-, and 660-km phase transitions	1.6, 4.3, −2.5	MPa K^{-1}
-	Density contrast at 410-, 520-, and 660-km phase transitions	7%, 3% 10%	-
$\Delta\eta_c$	Viscosity contrast between continents (C_1 = 1) and oceans (C_1 = 0)	free parameter	-
-	Density contrast between continents and surrounding mantle	100	kg m^{-3}
Dimensionless parameters			
Ra	Thermal Rayleigh number	5.72 × 10^7	-
$Ra_{ph(i)}$	Phase Rayleigh numbers for 410-km (i = 1), 520-km (i = 2), and 660-km (i = 3) phase transitions	5.34 × 10^7, 2.29 × 10^7, 7.63 × 10^7	-
$Ra_{ch(j)}$	Compositional Rayleigh number for continents (j = 1)	–2.31× 10^7	-
B_j (=$Ra_{ch(j)}/Ra$)	Buoyancy ratio for continents (j = 1)	–0.40	-
Q	Internal heating number	Time-dependent	-
ζ	Mantle/shell radius ratio	0.45	-
